# Glitches in the utilization of telehealth in pediatric rheumatology patients during the COVID-19 pandemic

**DOI:** 10.1186/s12969-020-00477-y

**Published:** 2020-10-15

**Authors:** Nayimisha Balmuri, Karen B. Onel

**Affiliations:** 1 Division of Pediatric Rheumatology, Hospital for Special Surgery, 535 East 70th Street, New York, NY 10021 USA; 2 Department of Pediatrics, NewYork Presbyterian/Weill Cornell Medical Center, 525 East 68th Street, New York, NY 10065 USA

**Keywords:** Technology, Telehealth, Mental health, Psychosocial health, Adolescent health, COVID-19

## Abstract

Telehealth is an extraordinary advancement of modern medicine. It has increased access to care for underserved populations and, in the case of pediatric rheumatology, has expanded the reach of a limited work force. During the Coronavirus Disease 2019 (COVID-19) pandemic, telehealth has radically changed the way healthcare workers have been able to deliver care while maintaining social distance. In addition to the infectious havoc of COVID-19, the pandemic has further altered the psychosocial milleu of our society which directly impacts the wellness and safety of our pediatric rheumatology patients. These psychosocial factors may be difficult to assess and triage solely using telehealth. The objective of this short review is to educate practitioners on the psychosocial concerns exacerbated by the COVID-19 pandemic and to discuss the possible hurdles in utilization of telehealth to care for our vulnerable patient population.

## Main text

The workforce shortage of pediatric rheumatology practitioners in the United States has been well documented with many states remaining underserved without full time pediatric rheumatologists or a severely limited work force [1]. This has led to the adaptation of shared care models utilizing adult rheumatologists or community generalists, the use of mid-level practitioners, and most recently, the introduction of telehealth. Despite demonstrating feasibility and acceptability in studies, the uptake of telehealth within pediatric rheumatology has been slow due to a variety of reimbursement related concerns, and technical and regulatory obstacles [2].

The Coronavirus Disease 2019 (COVID-19) pandemic is a global medical emergency that has radically changed the way healthcare workers have been able to deliver care. Beginning in March, the Centers for Medicare and Medicaid Services in the US have increased resources to expand telehealth services and eliminated former barriers and requirements that would usually require in-person visits [3]. Similar changes have been noted internationally in an effort to triage potential COVID-19 patients and to provide convenient access to routine care while allowing for social distancing [4]. What this rapid surge in utilization of telehealth has shown the medical community is that this modality is effective as an emergency response and is a feasible option for patient monitoring. Therefore, the integration of telehealth into standard healthcare practices is likely to become permanent. Despite a myriad of positives supporting utilization of telehealth there are important concerns to consider as pediatric practitioners, both general and subspecialists, strive to provide comprehensive patient and family centered care.

Most patients who are referred to a pediatric rheumatologist and are subsequently followed by this subspecialty over long periods of time fit into the chronically ill pediatric care model. These complex patients are at risk to receive fragmented care without proper coordination [5]. For this reason, the pediatric rheumatologist often acts as the medical home and has become an expert in patient centered care and the coordination of the multidisciplinary approach. Our current telehealth system is not uniformly built to accurately and carefully manage and coordinate the care for the chronically ill pediatric patient [6]. Within this population, the poor, the uninsured, and the minority children may be at increased risk for inferior coordination of services [5] and may also have decreased access to telehealth services.

Psychosocial risk evaluation, such as the validated HEADSS exam [7], are an important cornerstone of the pediatric visit and may be challenging to perform remotely. Parents are required to sign consent for televisits and may expect to be present for the entire exam making it difficult to discuss sensitive topics [8]. Depression and anxiety are common with pediatric rheumatologic diseases and are associated with poor adherence, quality of life, and long-term outcomes [9]. Studies have shown that pediatric patients with systemic lupus erythematosus and mixed connective tissue disease have a trend towards increased depressive symptoms and statistically significant increase of suicidal ideation compared to healthy pediatric controls [10]. Despite such high prevalence of mental health concerns within this population, there are poor rates of prior mental health treatment [11] and therefore an assumed lack of integration of mental health centered telemedicine. During the COVID-19 pandemic, it has been noted that children and adolescents with disabilities, already existing mental health problems, and low socioeconomic status have an increased risk of worsening suicidal ideation, depression, and anxiety [12, 13]. With sole utilization of telehealth, patients might be less likely to divulge worsening of acute on chronic mental health symptoms.

Patients with pediatric rheumatologic diseases have been noted to have increased risk of smoking, alcohol use, and illicit drug use and dependence [14, 15]. Due to the COVID-19 pandemic and need for social distancing, there has been a reported increase in substance use [16]. People who already have increased tendencies for use of substances as an aid to help with stress may be at increased risk for abuse due to the increased stress, anxieties, and feelings of isolation due to social distancing [17, 18]. Although there is availability of telehealth services for substance use disorders, there is a lack of providers in rural areas [19] and those who specialize in pediatrics. It can be difficult over a telehealth visit to provide an environment where a patient may feel comfortable speaking about these issues and to properly conduct a physical exam and read nonverbal cues which may increase a clinician’s suspicion for further evaluation for substance use disorder.

Importantly, the necessary social distancing measures needed to control the spread of COVID-19 are causing a “secondary pandemic” of neglect and abuse of children [20]. Similar patterns of increased violence towards children have been noted during periods of school closures associated with health emergencies such as Ebola [21].

children have been noted during periods of school closures associated with health emergencies such as Ebola [21]. In Illinois, it has been noted that child abuse hotline reports have dropped by 50% but it is believed to be due to children’s decreased access to mandated reporters, not a decrease in abuse [22]. Despite increasing screening for child abuse and neglect, it may be more difficult for children to disclose on a telehealth session if they are in the same environment with their abuser or are worried that they might be overheard [23].

## Conclusions

The American Academy of Pediatrics estimates that about a quarter of children with rheumatic disease live 80 miles or more from a pediatric rheumatologist [24]. Telehealth has done wonders for expanding the reach of pediatric rheumatology within the United States to care for our vulnerable population. During this current pandemic, we have seen the incredible capabilities of medical technology and the importance of telehealth in helping with triage, ensuring that patients have access to care, and aiding the medical community to economically withstand the financial impact of COVID-19. However, given the complicated psychosocial complexities of chronically ill children that are further exacerbated by the pandemic, hopefully we will be able to utilize telehealth as an adjunct to in-person multidisciplinary team approach visits and not as a sole means to care. As subspecialists but also medical home providers, it is our duty to not only focus on the medical pathology and pharmaceutical treatment of disease, but also the important comorbidities that impact the short term and long term well-being and safety of our patients.

## Data Availability

Not applicable.

## Abbreviation

COVID 2019: Coronavirus Disease 2019
